# Screening of potential novel candidate genes in schwannomatosis patients

**DOI:** 10.1002/humu.24424

**Published:** 2022-06-27

**Authors:** Cristina Perez‐Becerril, Andrew J. Wallace, Helene Schlecht, Naomi L. Bowers, Philip T. Smith, Carolyn Gokhale, Helen Eaton, Chris Charlton, Rachel Robinson, Ruth S. Charlton, D. Gareth Evans, Miriam J. Smith

**Affiliations:** ^1^ School of Biological Sciences, Division of Evolution, Infection and Genomics, Faculty of Biology, Medicine and Health University of Manchester Manchester UK; ^2^ Manchester Centre for Genomic Medicine, St Mary's Hospital Manchester University NHS Foundation Trust Manchester UK; ^3^ North East and Yorkshire Genomic Laboratory Hub St James's University Hospital Leeds UK

**Keywords:** candidate genes, *CDKN2A*, *CDKN2B*, *COQ6*, *DGCR8*, schwannomatosis screening

## Abstract

Schwannomatosis comprises a group of hereditary tumor predisposition syndromes characterized by, usually benign, multiple nerve sheath tumors, which frequently cause severe pain that does not typically respond to drug treatments. The most common schwannomatosis‐associated gene is *NF2*, but *SMARCB1* and *LZTR1* are also associated. There are still many cases in which no pathogenic variants (PVs) have been identified, suggesting the existence of as yet unidentified genetic risk factors. In this study, we performed extended genetic screening of 75 unrelated schwannomatosis patients without identified germline PVs in *NF2*, *LZTR1*, or *SMARCB1*. Screening of the coding region of *DGCR8*, *COQ6*, *CDKN2A*, and *CDKN2B* was carried out, based on previous reports that point to these genes as potential candidate genes for schwannomatosis. Deletions or duplications in *CDKN2A*, *CDKN2B*, and adjacent chromosome 9 region were assessed by multiplex ligation‐dependent probe amplification analysis. Sequencing analysis of a patient with multiple schwannomas and melanomas identified a novel duplication in the coding region of *CDKN2A*, disrupting both *p14ARF* and *p16INK4a*. Our results suggest that none of these genes are major contributors to schwannomatosis risk but the possibility remains that they may have a role in more complex mechanisms for tumor predisposition.

## 1 INTRODUCTION

Schwannomatosis comprises a group of autosomal dominant tumor predisposition syndromes characterized by the development of multiple schwannomas. The most common form is associated with the *NF2* gene, but at least two further genetically distinct forms exist. Causative variants for non‐*NF2*‐related schwannomatosis have been primarily identified in two genes; *SMARCB1* (SWI/SNF‐related, matrix‐associated, actin dependent regulator of chromatin, subfamily b, member 1) and *LZTR1* (leucine zipper like transcription regulator 1), both located in the chromosome 22q region although these variants only account for 30%−40% of sporadic cases and 70%−80% of familial cases (Evans et al., 2018; Hulsebos et al., 2007; Kehrer‐Sawatzki et al., 2017; Piotrowski et al., 2014). In addition, the majority of non‐*NF2*‐related schwannomatosis cases are sporadic (MacCollin et al., 2005), suggesting the existence of novel schwannomatosis variants and/or genes.

Previous studies have proposed a role for additional genes in the pathogenesis of schwannomatosis. Whole exome sequencing (WES) analysis of 10 Korean sporadic schwannomatosis patients, identified 26 variants of which 13 were predicted to be pathogenic from in silico analysis. One of these potentially pathogenic variants (PVs) was a missense change (NM_000077.4:c.85C>A; p.Ala29Ser) located in exon 1 of the cyclin dependent kinase inhibitor 2A (*CDKN2A*) gene, in the chromosome 9p21.3 region (Min et al., 2020).

*CDKN2A* encodes two proteins, p16INK4a and the alternatively translated p14ARF, both of which have a role in tumor suppression, through regulation of Rb and p53 pathways (Quelle et al., 1995; Zhang et al., 1998a,b). Loss of function of both *CDKN2A* and its tandemly linked gene *CDKN2B*, which encodes p15INK4b, another regulator of the Rb pathway (Hannon & Beach, 1994), have been implicated in a variety of cancers from central nervous system (CNS) tumors, including schwannomas (Ali et al., 2021; Almeida et al., 2008; Cancer Genome Atlas Research, 2008; S. Zhang et al., 1996) pancreatic cancer, renal cancer, and melanoma (Goldstein et al., 2006; Jafri et al., 2015; McNeal et al., 2015; Patel et al., 2020; Tu et al., 2018). Indeed, *CDKN2A* is one of the main susceptibility genes for familial melanoma with both point mutations and gene deletions implicated in pathogenesis (Goldstein & Tucker, 1997; Hussussian et al., 1994; Kamb et al., 1994; Pollock et al., 1998; Whiteman et al., 1997). In addition, a splicing variant in *CDKN2A* (NM_000077.4:c.151–1G>C), responsible for loss of p16INK4a and p14ARF has been reported in a number of families affected by multiple neoplasms, including nerve sheath tumors and melanomas (Petronzelli et al., 2001; Prowse et al., 2003; Sargen et al., 2016). Notably in the most recent of these reports, Sargen et al. (2016) observed that a number of nerve sheath tumors, across affected family members carrying the *CDKN2A* variant, presented features consistent with both schwannoma as well as neurofibroma histopathology.

Other proposed candidate genes for schwannomatosis include the coenzyme Q6, monooxygenase (*COQ6*) gene, and DGCR8 microprocessor complex subunit (*DGCR8*). There has been one report of a constitutional missense variant in exon 6 of *COQ6* (NM_182476.2: c.622G>C; p.Asp208His) segregating with disease in a schwannomatosis affected family (K. Zhang et al., 2014). More recently, a study identified a germline variant in exon 7 of *DGCR8* (NM_022720.6:c.1552G>A; p.Glu518Lys) in all affected members of a family with both euthyroid multinodular goiter (MNG) and schwannomatosis (Rivera et al., 2020). This variant, which was predicted to be pathogenic by a number of algorithms and by in silico models, was subsequently characterized to determine its role in disruption of micro RNA biogenesis. Furthermore, a recent analysis of 13 schwannomas from patients affected by schwannomatosis and MNG identified the p.Glu518Lys pathogenic variant in *DGCR8* as the only germline pathogenic variant in four of these tumors (Nogué et al., 2022). All 13 tumors were found to have loss of heterozygosity (LOH) in the chromosome 22q region containing *DGCR8*, *LZTR1*, *SMARCB1*, and *NF2*. For 5/13 tumors, all from the same individual, no other germline PVs in other schwannomatosis genes were identified and no somatic *NF2* variants were identified in 4/5 tumors resected from this patient. The authors propose a new model for schwannoma formation in which the inactivating mutation in *DGCR8* constitutes the first hit, whereas loss of the second *DGCR8* allele, along with *LZTR1*, *SMARCB1*, and *NF2* constitute hits 2, 3, 4, and 5. For some of these tumor, a sixth somatic hit was also seen in *NF2* in the remaining 22q allele.

The purpose of this study was to assess the contribution of variants in *COQ6*, *DGCR8*, *CDKN2A*, and *CDKN2B* to pathogenesis in a group of patients whose clinical features are consistent with schwannomatosis diagnosis, but for whom routine genetic analysis failed to identify PVs in the known schwannomatosis genes; *NF2*, *SMARCB1*, and *LZTR1*. These individuals were also negative for germline chromosome 22q11.2 deletions, to confirm schwannomatosis diagnosis.

## 2 MATERIALS AND METHODS

DNA extracted from lymphocytes of 77 schwannomatosis patients from 75 schwannomatosis families, from the local register at the Manchester Center for Genomic Medicine was used for analysis. Demographic data for our cohort is summarized in Table 1. All patients included in the study met current clinical diagnostic criteria for schwannomatosis (Evans et al., 2018). These patients had also previously undergone routine genetic screening from which no PVs in *NF2*, *SMARCB1*, or *LZTR1* were identified. Routine analysis for schwannomatosis consists of screening of the coding region of *NF2*, *SMARCB1*, and *LZTR1*, including 15 base pairs of intronic region at each side of exon−intron boundaries as well as part of the untranslated regions where PVs are known to occur. Forty‐two patient samples in our cohort were received in or after 2013 and have been screened using next generation sequencing (NGS) with a mean coverage of ×1000 for *NF2*, optimized for detection of mosaicism to a level of 5%. NGS analysis was also carried out at a read depth of ×350 for *SMARCB1* and *LZTR1* on 46/77 and 52/77 patients, respectively. For patients for whom *NF2* screening was carried out by Sanger sequencing only (34/77 individuals), one or more tumor samples were analyzed when available (10/34). Clinical genetic testing techniques used for screening of index cases are summarized in Supporting Information: Table S1. Genetic testing of two anatomically distinct tumor samples ruled out a diagnosis of mosaic NF2 for 2 of these patients. A summary of molecular testing of these tumors is presented in Supporting Information: Table S2. The remaining 32/35 whose samples were collected before 2013, and did not undergo NGS screening for *NF2* variants, were classified as schwannomatosis patients based on current clinical diagnostic criteria (Evans et al., 2018), but mosaic NF2 has not been excluded genetically. A summary of clinical details and results from clinical genetic testing for patients in our cohort is provided in Supporting Information: Table S2.

**Table 1:** Summary of demographics for cohort of schwannomatosis patients

% of total					
				Age at the time of genetic screening	
Male	Female	Familial	Sporadic	0−30	>30
57	43	12	88	19	81

The presence of copy number variants is also routinely assessed through multiplex ligation‐dependent probe amplification (MLPA) analysis of *NF2* (probe‐set P044; MRC Holland), *SMARCB1* (probe‐set P258; MRC Holland), and *LZTR1* (probe‐set P455; MRC Holland). Finally, LOH is assessed in tumor samples, when available, with *NF2* intragenic and flanking polymorphic microsatellite markers. Ethical approval for the study was obtained from the North West—Greater Manchester Central Research Ethics Committee (reference 10/H1008/74). Research based sample screening and analysis were carried out under ethics approval (reference 10/H1008/74) obtained from the North West 7–Greater Manchester Central Research Ethics Committee. Patient data from large clinical databases was anonymized for this study.

Primers were designed to target flanking sites at each side of exons for regions of *COQ6* (NM_182476.2), *DGCR8* (NM_022720.6), *CDKN2A* (NM_000077.4), and *CDKN2B* (NM_004936.3) and are listed in Supporting Information: Table S3. In addition, two intronic regions of *SMARCB1* known to harbor PVs for schwannomatosis (Piotrowski et al., 2021; Smith et al., 2020) were also screened. Sanger sequencing of amplicons was then carried out using BigDye™ Terminator v3.1 Cycle Sequencing Kit (Thermo Fisher Scientific) and an ABI 3100 automated sequencer (Applied Biosystems).

MLPA was performed for 70 of the 77 samples (for which DNA was available), using the SALSA MLPA kit, P419 probemix (MRC‐Holland) probe set from MRC Holland. Briefly, 100 ng DNA was used for the hybridization, ligation, and amplification of exon probes for control and test samples according to the manufacturer's instructions and analyzed on an ABI 3100 automated sequencer (Applied Biosystems).

In silico analysis was performed for all variants identified in our cohort. Potential pathogenicity of missense variants was assessed using REVEL v4.2 (Ioannidis et al., 2016) and BayesDel v4.2 (Feng, 2017). Nonsense and intronic variants were assessed using CADD v1.4 (Rentzsch et al., 2019). Maximum credible population allele frequency values (Whiffin et al., 2017) and, when applicable, constrain metrics from gnomAD v2.1.1 (Karczewski et al., 2020) and DECIPHER v11.9 (Firth et al., 2009) were also used to aid in classification of variants according to the American College of Medical Genetics and Genomics (ACMG) guidelines (Richards et al., 2015; Tavtigian et al., 2018) and the Association for Clinical Genomic Science (ACGS) best practice guidelines for variant classification in rare disease (Ellard et al., 2020). Potential splicing effects of variants were assessed using SpliceAI (Jaganathan et al., 2019), as well as MaxEntScan (Yeo & Burge, 2004), GeneSplicer (Pertea et al., 2001), NNSPLICE (Reese et al., 1997), EX‐SKIP (Raponi et al., 2011), and SpliceSiteFinder‐Like (Shapiro & Senapathy, 1987) as implemented in Alamut® Visual software.

## 3 RESULTS

No pathogenic or likely PVs were identified in *DGCR8*, *COQ6*, or *CDKN2B*. Bidirectional sequencing revealed a heterozygous single nucleotide duplication in exon 2 of *CDKN2A* (NG_007485.1:g.28291dup) in DNA from a patient with five nerve sheath tumors (two were considered hybrid tumors with high schwann cell content but some neurofibroma features). As the patient had no clinical features of NF1 and no vestibular schwannomas she was considered to have presumed schwannomatosis. Molecular analysis was carried out in one of the two independent hybrid tumors, found no PVs in *NF2*, *SMARCB1*, or *LZTR1* and no evidence for LOH for *NF2* markers. This patient also had family history of melanoma (affected paternal grandfather and uncle) and previously presented with two melanomas, which were a malignant melanoma Clark stage 3 and a superficial spreading melanoma in situ. The duplication identified in this patient disrupted both isoforms of *CDKN2A*, which code for p16INK4a (NM_000077.4:c.158dup; p.Met53fs) and p14ARF (NM_058195.3:c.201dup; p.Asp68Ter), respectively. This is similar to a previous report of a splicing variant in *CDKN2A* (NM_000077.4:c.151–1G>C) that resulted in inactivation of both gene isoforms (Sargen et al., 2016) and which was observed in DNA samples from three members of a family affected by melanoma and multiple nerve sheath tumors, some of which showed overlapping schwannoma and neurofibroma features.

**Figure 1:**
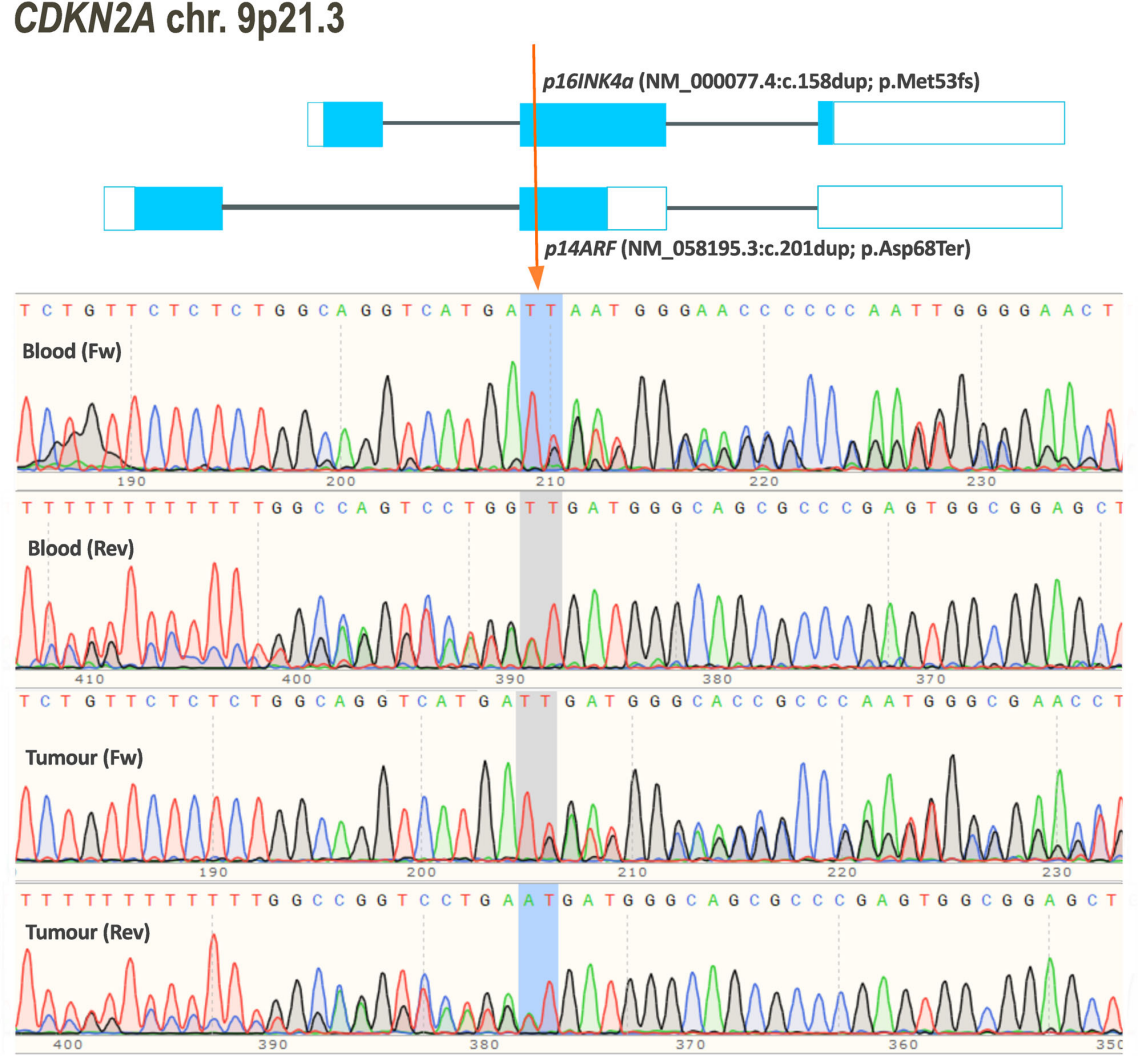
Duplication in *CDKN2A* in a schwannomatosis patient also diagnosed with melanoma. Schematic representation of both isoforms of *CDKN2A*, indicating the position of a single base pair (T) duplication that results in a frame shift for both proteins, p16INK4a (NM_000077.4:c.158dup; p.Met53fs) and p14ARF (NM_058195.3:c.201dup; p.Asp68Ter). The duplication was identified by bidirectional sequencing of a lymphocyte derived DNA sample from a schwannomatosis patient who was also diagnosed with melanoma. Additionally, a schwannoma sample was available for sequencing analysis, which confirmed the presence of this variant in the tumor.

The variant we identified in *CDKN2A* has not been reported previously but it is located in a highly conserved region and has been classified as pathogenic based on ACMG and ACGS guidelines (Figure 1). To investigate this duplication in schwannomatosis, we screened the coding sequence of *CDKN2A* in one schwannoma sample from this same patient. The NM_000077.4:c.158dup/NM_058195.3:c.201dup was also present in heterozygous form. No other variants were identified, except previously reported polymorphisms. Previous analysis of this tumor sample revealed no evidence of 22q involvement. The variant was inherited from her unaffected father. Her paternal grandfather and uncle both had a history of melanoma; however DNA was not available from these family members for molecular testing.

MLPA analysis was carried out in 70 people for whom high quality DNA was available. No copy number abnormalities were detected in *CDKN2A* or *CDKN2B* for any of the samples analyzed. A reproducible decrease in signal of a single control probe for the transmembrane channel like 1 (*TMC1*) gene on chromosome 9q21.13, was seen in a patient with thyroid cancer and schwannomatosis. Sequencing of the probe region found no known polymorphisms, suggesting the possibility of hemizygosity. Mutations in the *TMC1* gene have been associated with congenital and progressive hearing loss (Kurima et al., 2002) but no links to thyroid disorders or predisposition to nervous system tumors have been established. However, the significance of this result remains unclear. Previous analysis of schwannoma DNA identified LOH on chromosome 22, but subsequent MLPA analysis for *CDKN2A* failed for this sample.

## 4 DISCUSSION

The predominant model of inheritance for familial non‐*NF2*‐related schwannomatosis is a dominant model, similar to that of NF1 and *NF2*‐related schwannomatosis. However, whilst routine genetic analysis is able to identify around 92%−95% of variants responsible for familial cases for NF1 and *NF2*‐related schwannomatosis, the proportion of familial non‐*NF2*‐related schwannomatosis cases that can be attributed to known variants is much lower (70%−80%). This proportion is even lower for sporadic schwannomatosis cases (30%−40%) (Evans et al., 2018; Kehrer‐Sawatzki et al., 2017). The genetic architecture of non‐*NF2*‐related schwannomatosis appears to be more complex than that of NF1 and *NF2*‐related schwannomatosis with constitutional *SMARCB1* PVs contributing in much higher proportion to familial schwannomatosis than they do to sporadic cases, whereas germline *LZTR1* PVs seem to contribute similarly to familial and sporadic cases (Evans et al., 2018). This has prompted increasing efforts to identify novel PVs for schwannomatosis, some of which might turn out to be located in hitherto undiscovered schwannomatosis loci.

Many of these efforts have benefitted from advances in technologies for genomic analysis. WES of DNA from both sporadic and familial schwannomatosis samples have implicated a number of genes and variants of interest (K. Zhang et al., 2014; Min et al., 2020; Rivera et al., 2020). However, the extent to which these variants can be considered causative for schwannomatosis has proved to be harder to determine. In some cases, such as the recently reported germline variant in *DGCR8* (NM_022720.7:c.1552G>A; p.Glu518Lys), functional characterization has provided strong evidence of the pathogenic nature of the variant. Additionally, clinical features of individuals carrying this variant prompted the authors to conclude that the variant might define a novel syndrome, characterized by the cooccurrence of schwannomatosis and familial MNG. Of note, only one of the schwannomatosis patients in our cohort had a thyroid related comorbidity, which was not classified as familial and no *DGCR8* variant was identified in this patient.

In contrast to *DGCR8*, evidence supporting the involvement of variants in *CDKN2A* and *COQ6* in schwannomatosis pathogenesis is less conclusive. Previous variants reported as potentially associated with schwannomatosis for these genes (K. Zhang et al., 2014; Min et al., 2020) have not been fully characterized, so the mechanisms through which they might contribute to disease remain unclear.

In the case of *COQ6*, there is need for more exhaustive functional analysis to establish a plausible mechanism through which dysfunction of *COQ6* might lead to schwannomatosis, particularly in the absence of evidence for bi‐allelic inactivation of this gene in tumor tissue from affected members of the family in which the variant was originally identified. Indeed, questions have been raised about the role of this variant as a causative variant for schwannomatosis (Trevisson et al., 2015), since there were other variants that were identified by the same study that originally reported the *COQ6* variant, but that were not deemed of interest by the authors. To date, no other studies have identified *COQ6* variants in schwannomatosis patients, including our present study.

A link between malignant melanoma and nervous system tumors has been established by a number of studies. Particularly, germline whole gene deletions of *CDKN2A* and *CDKN2B* are known to be associated with familial syndromes predisposing to malignant melanoma as well as other nervous system tumors, including meningioma, astrocytoma, and schwannoma (Azizi et al., 1995; Bahuau et al., 1997, 1998; Chan et al., 2017; Kaufman et al., 1993). At the somatic level, inactivation of *CDKN2A* and *CDKN2B* has been identified as an important feature in a number of tumors, most notably melanomas and tumors in the CNS (Boström et al., 2001; Casula et al., 2019; Ghasimi et al., 2016; Gonzalez‐Zulueta et al., 1995; McNeal et al., 2015; Rousseau et al., 2003). Interestingly, loss of CDKN2 proteins in meningiomas has been established as an important consideration for tumor classification and, in some cases, a determinant of tumor progression (Goutagny et al., 2010; Suppiah et al., 2019). This raises the possibility for a more prominent role of variants in *CDKN2A* and *CDKN2B* genes as modulators of clinical phenotypes. The role of *CDKN2A* dysfunction in the pathology of schwannomatosis has not been established but some clues may emerge from the study of the mechanisms involved in transformation of neurofibromas into malignant peripheral nerve sheath tumors (MPNSTs) resulting from bi‐allelic inactivation of *CDKN2A* (Chaney et al., 2020; Magallón‐Lorenz et al., 2021; Nielsen et al., 1999). These mechanisms appear to be relevant not only to MPNSTs tumor progression but also to multiple malignant and benign tumor predisposition (Sargen et al., 2016). It is therefore possible that the in *CDKN2A* we report here might be contributing to a similar complex syndrome.

The absence of germline PVs in *DGCR8*, *COQ6*, or *CDKN2B* within a group of clinically well characterized schwannomatosis patients suggests that none of these genes is likely to be a major contributor to schwannomatosis pathogenesis on its own, although the possibility remains that they may have a role in complex clinical phenotypes. There is also a possibility that at least a proportion of the schwannomatosis cases that remain genetically unexplained might be caused by a variant within *NF2*, *SMARCB1*, or *LZTR1* that has been missed by routine diagnostic methods. This could help explain the fact that previous studies using whole genome sequencing of schwannomatosis patients have either been unable to identify novel candidate genes (Hutter et al., 2014) or have reported potentially PVs in a number of loci, which have not been validated (K. Zhang et al., 2014; Min et al., 2020). Indeed a deep intronic PV, NM_003073.5:c.795+1498C>T, leading to the inclusion of a cryptic exon and a truncated product, has previously been reported in intron 6 of *SMARCB1* (Smith et al., 2020). In addition, a recent deep massive parallel sequencing study of 35 schwannomatosis cases (Piotrowski et al., 2021) reported two novel deep intronic variants in intron 4 of *SMARCB1* (NM_003073.5:c.500+883T>G and NM_003073.5:c.500+887G>A). Both of these variants were further characterized by means of RNA analysis and demonstrated to result in retention of part of intron 4 and a truncated transcript. We have screened these intronic regions in our cohort and found that none of our patients is a carrier for any of these three variants. The variants in intron 4 were covered by our clinical NGS panel, but deep‐intronic regions are not typically scrutinized for diagnostic purposes. The intron 6 variant was not captured by the panel. Furthermore, limitations of some of the techniques used in the past for genetic molecular testing might mean that for some individuals, low level mosaic variants in *NF2* have been missed. This in turn may result in misdiagnosis of mosaic NF2 cases as non‐*NF2*‐related schwannomatosis, particularly for cases where there is limited availability of tumor tissue (Evans et al., 2018; Kehrer‐Sawatzki et al., 2018). The current use of high read depth NGS analysis in clinical genetic testing has improved the rate of detection for mosaic PVs in *NF2* (Contini et al., 2015; Evans et al., 2020; Louvrier et al., 2018), however reanalysis of patient samples is not always possible. Future efforts to find novel PVs for schwannomatosis might be greatly aided by a similar approach to the one used by Piotrowski and colleagues, which involved deep sequencing of the full gene region of *NF2*, *SMARCB1* and *LZTR1*, which will help detect noncoding PVs that are difficult to identify by standard NGS panels or WES.

In addition, there is accumulating evidence for PVs contributing to schwannomatosis combined with other conditions has been set by the case for families affected by more than one condition in which a particular variant is observed to cosegregate with disease, such as the previously described variant in *DGCR8* (Rivera et al., 2020) or for families affected by *CDKN2A*‐associated melanoma, who also have an increased risk for other cancers present (Goldstein et al., 2006). This is also the case for people affected by melanoma along with nervous system tumors due to deletion of both *CDKN2A* and *CDKN2B*, with suggestions that inactivation of *CDKN2A/B* genes might be responsible for the melanoma phenotype, while loss of adjacent genes might contribute to the development of other neoplasms (Chan et al., 2017). The presence of schwannomas in our melanoma patient and the retention of the single base duplication in *CDKN2A* in tumor DNA suggest that inactivation of *p14ARF* and *p16ink4a*, may be enough for schwannoma formation. The presence of additional factors contributing to risk for both conditions has also been explored in previous studies. One intriguing possibility is the potential effect of noncoding elements on the observed phenotype. One of these elements, a long noncoding RNA (*CDKN2B‐AS1*) spanning the two exons of *CDKN2B*, was discovered by a study aimed to determine the size of a 9p21 deletion in a large family affected by melanoma‐astrocytoma syndrome (Pasmant et al., 2007). The authors suggest, *p14ARF* and *CDKN2B‐AS1* might share a promoter, a fact supported by their discovery of a significant correlation in transcript levels of *CDKN2B‐AS1* with those of *p14ARF*, *p16INK4a*, and *CDKN2B* in healthy tissue as well as breast tumor samples and NF1‐associated tumor samples.

Exploration of the possible interactions of potential candidate genes for schwannomatosis with the known causative genes, might provide some insight into the type and location of novel PVs in cases where no variants in *SMARCB1*, *LZTR1*, or *NF2* have been identified. Furthermore, functional characterization of known causative variants for schwannomatosis will undoubtedly advance our understanding of potentially new mechanisms of disease in schwannomatosis, particularly for variants located in noncoding regions of both *SMARCB1* and *LZTR1*. This in turn might lead to elucidation of important correlations of these genes with other loci, and ultimately to an increased ability for accurate diagnosis and classification of schwannomatosis cases based on their clinical and molecular features.

## CONFLICT OF INTEREST

The authors declare no conflict of interest.

## Supporting information

Supporting information.

Supporting information.

## Data Availability

The data that support the findings of this study are available from the corresponding author upon reasonable request. An entry has been made for the *CDKN2A* NM_000077.4:c.158dup variant in the LOVD database and can be found here: https://databases.lovd.nl/shared/variants/0000839293#00004876
