# Supplementary material for: Screening of potential novel candidate genes in schwannomatosis patients
Source: Hum Mutat. 2022 Jun 27;43(10):1368–76. doi: 10.1002/humu.24424 (PMC9540472; doi:10.1002/humu.24424)
Supplement: Supplementary file 1 — Supporting information. [file HUMU-43-1368-s001.pdf]

Supplementary materials

Screening of potential novel candidate genes in schwannomatosis patients.

Cristina Perez-Becerril[1,2], Andrew J. Wallace[2], Helene Schlecht[2], Naomi L. Bowers[2], Philip T. Smith[2], Carolyn Gokhale[2], Helen Eaton[2], Chris Charlton[2],Rachel Robinson[3], Ruth S Charlton[3], D. Gareth Evans[1,2], Miriam J. Smith[1,2].

Supporting Table S1. Clinical genetic testing summary.

	No. of Index Cases		
	Screening for Germline PVs		
	NGS	Sanger sequencing	MLPA
NF2	43	32	75
SMARCB1	47	28	75
LZTR1	53	22	75

Supporting Table S3. Primer sequence for amplification of exonic regions of candidate genes and two intronic regions in *SMARCB1*.

Gene	Exon/Intron	Sequence (5'-3')
DGCR8	E2Fa	CAATGTGGCCAGCTTGACTAAG
DGCR8	E2Ra	CTGTGTACAGCACCTTCCTGTC
DGCR8	E2Fb	CCTGACCTTAAGTTGCTTAAGG
DGCR8	E2Rb	GCTGCAATTCCCAAATCTCTC
DGCR8	E3F	GTTCTCAGGAATGCTGTTGAG
DGCR8	E3R	GTCCTGTCCTCAAACATGCC
DGCR8	E4F	GTACCCAACAATTTCTTGTGC
DGCR8	E5R	CAACAGTGGAGGTGCACAG
DGCR8	E6F	GAAATAGTGTCATGTGGCCTG
DGCR8	E7R	CTGACCAAAGTTACACCTTGTG
DGCR8	E8F	CTGACTGCGCCTTGTCTGTC
DGCR8	E8R	CACTTGTCACCACTGCTCAG
DGCR8	E9F	CTGTCACTGAAGTGCTGTCTC
DGCR8	E9R	CAGCTCACACTAACAGGGCAG
DGCR8	E10F	CTAGGCGAGCTCACAAGAAC
DGCR8	E11R	GACAATGCCAGGGCAACAG
DGCR8	E12F	GAGTGCTGCCTATTCCTGTAG
DGCR8	E12R	GCACAGAGCCTCAGGAATAC
DGCR8	E13F	CTGTTTCGTGTCTGCCAGAC
DGCR8	E13R	GAAGGAGGATCCAAGCATTTC
DGCR8	E14F	CTAGCCTGGCATCACAAGC
DGCR8	E14R	CATGGCTGTCCCTGTACAC
COQ6	E1F	GAGGCAAGGTTCGTTTTCC
COQ6	E1R	CACTTTTGACCCTAATGGACC
COQ6	E2F	GGAGAAGTCTTACAGTTGTGGG
COQ6	E2R	GAGCTCCCTCCTTGTCAGATG
COQ6	E3F	GTAACAGGATGGAGGGACAAGG

Supporting Table S2 (continued).

COQ6	E4R	CAGAGGATTTCTTTATGGTGG
COQ6	E5F	GAGTTATCTCTGTGGCTTCTGTC
COQ6	E5R	CGATGAGACCATCTCCACTTG
COQ6	E6F	CACACAACAATCAGAGCTGGAG
COQ6	E7R	GCAGATTCCCTTGAAACCAG
COQ6	E8F	CTGGTTTCAAGGGAATCTGC
COQ6	E8R	GAGATTGCACAGTGAGCCAAG
COQ6	E9F	CAGTGTAGGCAAGTTGGGTAG
COQ6	E10R	TGTCCACTCCCTCTTGCTACT
COQ6	E11F	CTATGGGGAAGTATCCAGAGGTC
COQ6	E11R	CAGGATAATCACTTGAACCTGG
COQ6	E12	CAAGGACACTTGGGAAGAATAC
COQ6	E12	GGTCTGGCTAATATACGCTCCTC
CDNK2A	E1F	GTTCCGAGATCTTGGAGGTC
CDNK2A	E1R	CTGTGTGAAGGGAGGTCCAG
CDNK2A	E2F	CGACAGCTCCGCAGAAGTTC
CDNK2A	E2R	CTGAGGCAAGACCGGAGACTG
CDKN2A	E3F	GTACATGCACGTGAAGCCATTG
CDKN2A	E3R	CTTCGGTGACTGATGATCTAAG
CDNK2B	E1F	AGGACGACGGGAGGGTAATG
CDNK2B	E1R	CTACATCGGCGATCTAGGTTC
CDNK2B	E2F	ACACCAGACATCAGAGACCTG
CDNK2B	E2R	CAGACAGGCTTGCAGGCTTAC
TCM1	E15F	CTCAGATTTAGTTTACATT
TCM1	E15R	GTTAACTGTAAGGGCAGGATAG
SMARCB1	I4F*	-
SMARCB1	I4R*	-
SMARCB1	I6F	CACCATATTTGTCAGTCTGG
SMARCB1	I6R	GGACAAATACTGCATGATTCC

*This region was interrogated using NGS data.
